# Traumatic Neuroma as a Rare Complication of "Vel" Piercing

**DOI:** 10.7759/cureus.75456

**Published:** 2024-12-10

**Authors:** N R Shrinivaasan, Sharada T Rajan, Suganya Ramalingam, Venkata Chalapthi S, Thamizhchelvan H, Shalini S G

**Affiliations:** 1 Orthodontics, Dhanalakshmi Srinivasan Dental College, Perambalur, IND; 2 Oral Pathology and Microbiology, Sri Ramachandra Dental College and Hospital, Sri Ramachandra Institute of Higher Education and Research, Chennai, IND; 3 Oral and Maxillofacial Surgery, Dr. Shrini Dental Care, Perambalur, IND

**Keywords:** bilateral swelling, body piercing, nerve fascicles, traumatic neuroma, "vel"

## Abstract

Body piercing has become popular among people of all ages, inspired by religious convictions, personal aesthetics, and cultural influences. Piercings in the oral cavity frequently involve the lip, buccal mucosa, frenulum, and tongue with needles, hooks, and rings. However, these piercings might cause long-term problems to both the hard and soft tissues of the mouth. Our case report emphasizes the relevance of comparing clinical and histological results to provide an accurate diagnosis and appropriate treatment.

## Introduction

Traumatic neuroma is otherwise referred to as amputation or post-traumatic neuroma. This occurs following an accident or trauma to the soft tissues which can inadvertently result in the severance of the adjacent nerve fibers. Following such trauma, a reparative process occurs that can enable reinnervation. In case of any impediments, the nerve proliferates in a haphazard manner, thereby resulting in a mass of unorganized nerve tissues constituting traumatic neuroma. Occasionally, certain religious or cosmetic practices, such as piercings of the various parts of the body including the oral cavity, can result in traumatic neuroma.

The development of traumatic neuroma is a rare but interesting condition that can arise following trauma to the nerve tissue. Traumatic neuromas are characterized by the formation of a disorganized mass of nerve fibers, which can cause symptoms such as pain, numbness, and paresthesia [1].

A comprehensive understanding of the clinical presentation, combined with histopathological examination, is essential for differentiating traumatic neuroma from other conditions that may present with similar symptoms [2]. Our case report highlights the importance of correlating clinical and histopathological findings to establish an accurate diagnosis of a traumatic neuroma. 

## Case presentation

**Figure 1:**
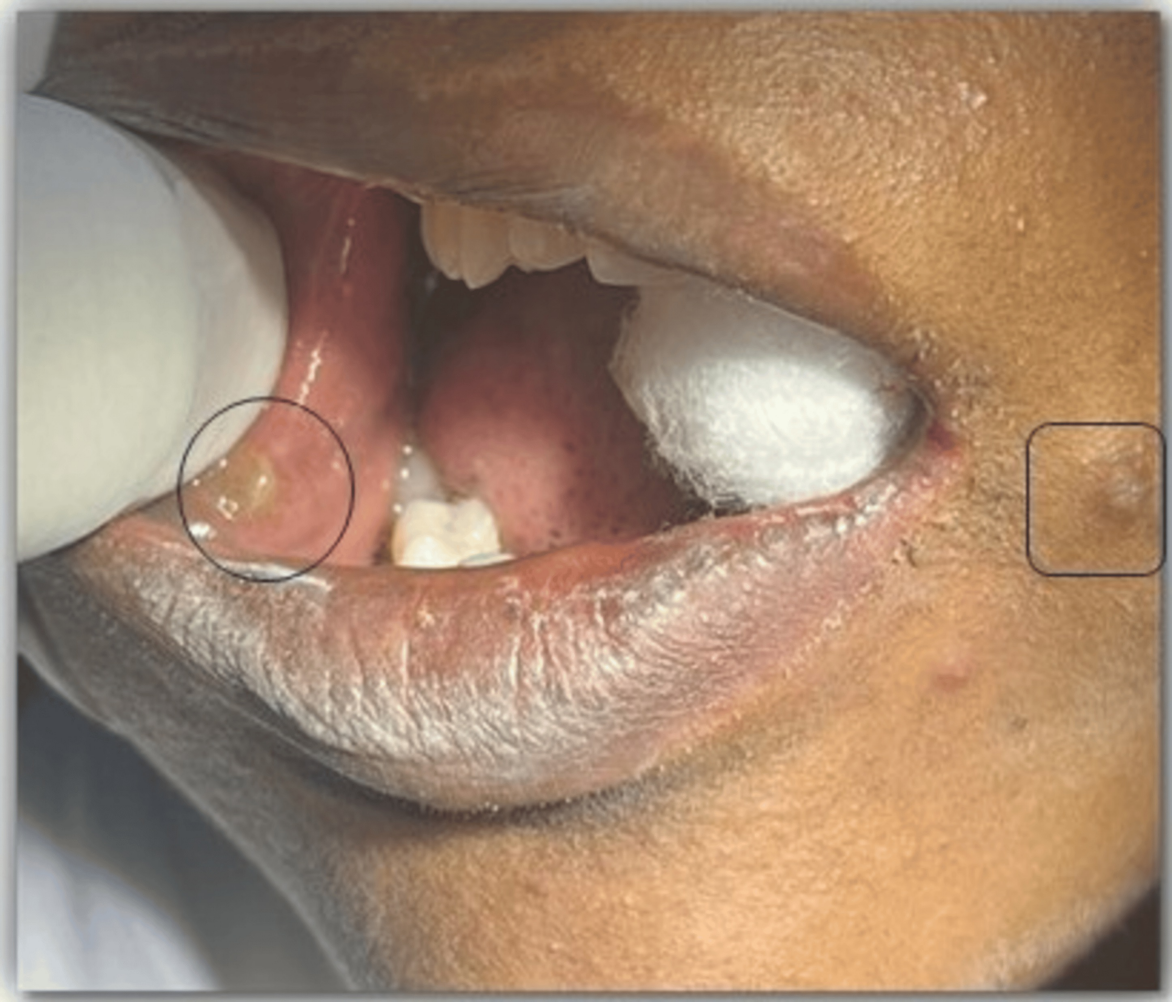
Bilateral firm swelling in the buccal mucosa

**Figure 2:**
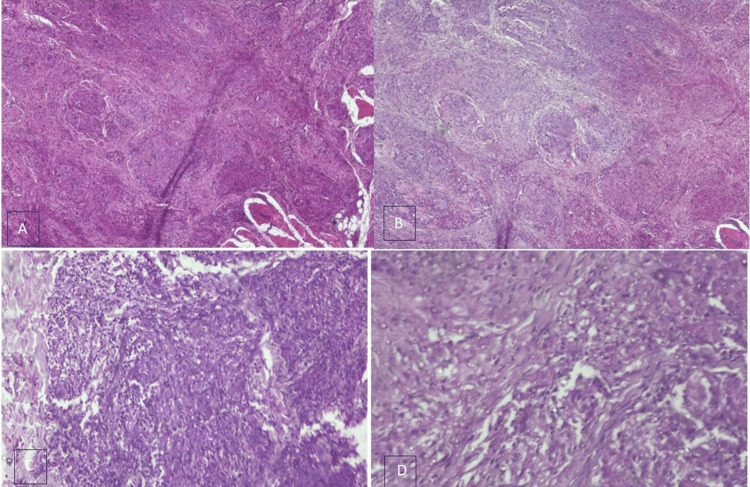
H&E section shows spindle-shaped nerve fascicles with prominent nuclei in the background of collagen fibers and entrapped muscle fibers along with areas of multinucleated giant cells and ganglion cells H&E: hematoxylin and eosin

Traumatic neuromas are reparative lesions, which is mainly due to the result of trauma. Although relatively uncommon, oral traumatic neuromas are one of the more common entities of head and neck traumatic neuromas. A 31-year-old female patient was referred from a private clinic to the Department of Oral Pathology and Microbiology, Sri Ramachandra Dental College. The patient complained of bilateral swelling in the buccal mucosa for the past few years. The patient had a history of piercing of a religious spear known as "Vel" in the colloquial language (seven years ago). A papulonodular eruption, whitish-grey, was present bilaterally in the buccal mucosa closer to the commissure of the lips. On examination, a bilateral firm diffuse swelling roughly measuring about 3 cm was palpable in the buccal mucosa (Figure 1). It was not associated with pain or discharge. The provisional diagnosis was given as traumatic fibroma. An incisional biopsy was performed which showed spindle-shaped nerve fascicles with prominent nuclei in the background of collagen fibers and entrapped muscle fibers. Areas of multinucleated giant cells along with areas of ganglion cells were seen (Figure 2A-2D). A histopathological diagnosis of traumatic neuroma was given. Post-histopathological diagnosis, surgical resection of the lesion in toto with margins of 2 mm was achieved with precision engaging diode laser of 10 W and 980 nm. Laser resection enabled clear margins with marked control of bleeding during the procedure that enabled uneventful healing.

## Discussion

Traumatic neuroma is considered as hyperplastic, reactive lesions of the peripheral nerve tissue and not related to the true neoplasm which usually presents with pain, burning, or paresthesia along with a history of injury or trauma in a particular site [3,4]. The occurrence of traumatic neuroma in the oral cavity is extremely rare; however, it has been seen in the region of the tongue, lower labial mucosa, and mental foramen as a solitary nodule [4,5]. Traumatic neuroma, also known as amputation neuroma, is a non-neoplastic proliferation of nerve response towards an injury. The severed nerve ends re-establish its continuity by quickly growing axons from the proximal to distal stump via the proliferating Schwann cells. However, if nerve endings are not closely apposed or if the distal stump is disorganized, proximal nerve neuroma will proliferate. Supernumerary digits that suffer auto-amputation in utero are a rare kind of traumatic neuroma.

Traumatic neuromas can exhibit clinically with a wide variety of characteristic features in the head and neck region and also in other parts of the body, which usually confuses clinicians. Traumatic neuromas are reactive lesions that occur after the resection or injury to the nerve bundles, with the main occurrence in women. It is otherwise known as amputation neuromas [5]. These lesions often occur immediately after any traumatic experiences or appear weeks or months later [6]. This is in concordance with our case report.

These lesions are caused by the reactive growth of nerve fibers as a result of insufficient recovery following peripheral nerve injury. The proximal section of the injured nerve regenerates and restores nerve distribution by sending axons to the distal segment. The interference of scar tissue with this process causes the regenerated fibers to twin back on themselves, resulting in a mass comparable to a ball of worms with a smooth surface submucosal nodule. The lesion is slightly more predominant in women which causes soreness and a burning sensation in the affected site.

Clinically, it presents as a firm nodule occasionally painful or tender and may have a chance for strangulation of proliferating nerve by scar tissue, trauma, or local infiltration. Grossly, it presents as circumscribed greyish-white nodules seldom more than 5 cm in diameter. Histopathologically, there is a presence of random proliferation of nerve fascicles admixed with axons with their investitures of myelin, Schwann cells, and fibroblasts. Fascicles may be seen myelinated than the parent nerve, embedded in a collagen background. Histopathologically moderately loose fibrovascular stroma mixed with interlacing randomly arranged nerve fibers is evident. Nerves proliferate furiously while confined inside thick and fibrous scar tissue. The Luxol quick blue stain will indicate which fibers are myelinated and which are not.

Various complications arise from body piercing. In various spiritual practices, followers pierce their oral cavity and body parts with metal rods, known as "kavadi," to reach spiritual purity. In our case, a patient used a "Vel" rod, which is oval-shaped with a pointed tip. When metal reacts with saliva, it can corrode easily, thereby releasing metal ions and causing hypersensitivity and contact allergies [7].

Ludwig's angina is an inflammation of the connective tissue of the floor of the mouth and neck which is an uncommon complication of an oral piercing infection. It is a condition that can be fatal. The histopathological differential diagnosis for this case includes palisaded encapsulated neuroma (PEN), schwannoma, and neurofibroma. The presence and identification of damaged nerves rule out neurofibroma. PEN is predominant in women, exclusively in the skin, with more circumscribed and orderly arranged nerve fascicles. Treatment attempts to reoppose the ends of the severed nerve result in regeneration in an orderly fashion. Neuromas can be removed when symptomatic. Treatment aspects of this disease include simple excision. When performed using diathermy, recurrence of this tumor is very unlikely as it prevents the leaving behind of any residual tissue and is, therefore, the recommended choice of treatment [4,7]. In the current case, resection with laser also proved to be advantageous with no sutures that accentuated the formation of pink granulomatous tissue aiding complete healing.

## Conclusions

Body piercing-related traumatic neuroma is a rare but significant complication that can occur in the oral cavity. Clinicians must notice this illness and include it in their differential diagnosis when evaluating patients with nerve injury symptoms. This case report serves as a crucial reminder to clinicians to raise awareness among patients about the potential risks and implications associated with religious piercing beliefs. Healthcare providers need to educate patients about the possible complications that can arise from these practices, including the development of traumatic neuroma.
